# Missing Value Imputation of Wireless Sensor Data for Environmental Monitoring

**DOI:** 10.3390/s24082416

**Published:** 2024-04-10

**Authors:** Thomas Decorte, Steven Mortier, Jonas J. Lembrechts, Filip J. R. Meysman, Steven Latré, Erik Mannens, Tim Verdonck

**Affiliations:** 1 Department of Mathematics, University of Antwerp-imec, Middelheimlaan 1, 2000 Antwerp, Belgium; thomas.decorte@uantwerpen.be; 2 IDLab, Department of Computer Science, University of Antwerp-imec, Sint-Pietersvliet 7, 2000 Antwerp, Belgium; steven.mortier@uantwerpen.be (S.M.); steven.latre@uantwerpen.be (S.L.); erik.mannens@uantwerpen.be (E.M.); 3 Plants and Ecosystems, Department of Biology, University of Antwerp, Universiteitsplein 1, 2610 Antwerp, Belgium; jonas.lembrechts@uantwerpen.be; 4 Geobiology, Department of Biology, University of Antwerp, Universiteitsplein 1, 2610 Antwerp, Belgium; filip.meysman@uantwerpen.be

**Keywords:** missing data, imputation, wireless sensor networks, environmental monitoring, time series

## Abstract

Over the past few years, the scale of sensor networks has greatly expanded. This generates extended spatiotemporal datasets, which form a crucial information resource in numerous fields, ranging from sports and healthcare to environmental science and surveillance. Unfortunately, these datasets often contain missing values due to systematic or inadvertent sensor misoperation. This incompleteness hampers the subsequent data analysis, yet addressing these missing observations forms a challenging problem. This is especially the case when both the temporal correlation of timestamps within a single sensor and the spatial correlation between sensors are important. Here, we apply and evaluate 12 imputation methods to complete the missing values in a dataset originating from large-scale environmental monitoring. As part of a large citizen science project, IoT-based microclimate sensors were deployed for six months in 4400 gardens across the region of Flanders, generating 15-min recordings of temperature and soil moisture. Methods based on spatial recovery as well as time-based imputation were evaluated, including Spline Interpolation, MissForest, MICE, MCMC, M-RNN, BRITS, and others. The performance of these imputation methods was evaluated for different proportions of missing data (ranging from 10% to 50%), as well as a realistic missing value scenario. Techniques leveraging the spatial features of the data tend to outperform the time-based methods, with matrix completion techniques providing the best performance. Our results therefore provide a tool to maximize the benefit from costly, large-scale environmental monitoring efforts.

## 1. Introduction

Over the last decade, major advances in wireless communication technology, microelectronics, and (big) data analytics have caused a significant increase in the application of Wireless Sensor Networks (WSNs) [1,2]. A WSN comprises a network of many spatially distributed sensors that monitor certain parameters of a physical system and engage in wireless data communication. The WSN is made up of sensor nodes, sometimes also called sensor motes, which are essentially microcomputers with the ability to collect data, process these data internally, and finally transmit these data to a centralized location. WSNs have numerous applications in different fields, including environmental monitoring, health monitoring, logistics, and smart cities [3,4]. With the increasing use of WSNs, there is a growing demand for performant data analysis techniques capable of handling the vast volumes of collected data.

An important challenge within WSN research concerns missing value imputation for the extensive spatiotemporal datasets that are generated. Unavoidably, networks tend to lose readings from sensors for reasons that are difficult or impossible to anticipate, such as sensor failure due to power depletion, network outages, and communication errors, but also destruction due to storms or vandalism [5]. These missing readings can have important consequences for real-time monitoring, for example, in an emergency setting. Likewise, environmental monitoring applications relying on WSN data can suffer from missing data, which might lead to delayed or incorrect responses to environmental changes. Additionally, missing values can weaken the reliability of sensor data and increase the difficulty of sensor calibration. Finally, incomplete data can also compromise the performance of subsequent modeling and statistical analysis, which may result in biased conclusions or inaccurate predictions. A concrete example can be found in environmental research, where a WSN is commonly leveraged to measure variables such as temperature, humidity, atmosphere pressure, and sunlight, among others. Despite the wealth of data collected by sensor nodes, they often exist in raw form. Analytical tools commonly employed in such fields, such as support vector machines, principal component analysis, and singular value decomposition, face limitations when confronted with datasets containing missing data. Consequently, addressing the issue of missing data in these datasets presents a significant hurdle, impacting the efficacy of analyses and hindering the ability to draw meaningful conclusions [6].

The objective of this study was to evaluate the performance of missing value imputation techniques on a dataset generated by a WSN for environmental monitoring. To this end, we employ a unique dataset that originated from one of the largest citizen science projects to date involving Internet of Things (IoT) monitoring. Throughout the summers of 2021 and 2022, 4400 citizens within the region of Flanders (Belgium) installed IoT sensors in their gardens to measure the temperature and soil moisture at a high temporal frequency (every 15 min). The goal of this citizen science project, called Curieuze-Neuzen in de Tuin; Nosy Parkers in the Garden (CNidT), was to gain insight into how garden ecosystems can provide cooling for climate adaptation and mitigate the impacts of extreme weather events like heat waves. In projects like CNidT, missing values in the sensor time series are undesirable, both from a scientific and from a citizen perspective. From a scientific perspective, the data incompleteness reduces the power of the ensuing statistical analysis, which here aimed to uncover the factors that drive local garden cooling during extreme weather events. Likewise, data incompleteness was also highly unwanted from the citizen perspective: participating citizens were updated daily through personal dashboards, while society at large was informed through real-time maps on the website of a national newspaper. However, missing values were common in the recorded time series due to a combination of random sensor failure (e.g., battery problems), failed data transfers (e.g., due to network outages), and errors made by the citizens (e.g., destruction or damaging of the sensor). For these reasons, the dataset from the CNidT project was especially suitable as a case study for missing value imputation in WSN data. The CNidT dataset is an integral component of the SoilTemp project, which is a publicly available database outlined in Lembrechts et al. (2020) [7]. This extensive database comprises data from 7538 temperature sensors spanning 51 countries and encompassing diverse biomes. The primary objective of the SoilTemp project is to enhance the global comprehension of microclimates and to address discrepancies between existing climate data and the finer spatiotemporal resolutions pertinent to organisms and ecosystem dynamics [7].

Given that missing data within WSNs pose a fundamental challenge, the development of methods capable of imputing these missing values represents an active area of research. Within our study, several imputation approaches were evaluated to analyze their performance. An overview of all considered approaches and their imputation strategies is given in Table 1. A first approach involves techniques that take advantage of the temporal correlation between data, thus imputing missing values for a given sensor using the available data of that same sensor at different time steps. Evaluated methods for this approach include mean and linear spline imputation [8]. A second class of techniques utilizes spatial correlation to impute values, focusing on data from other sensors in the network at the same time step to impute the missing values of one sensor. Evaluated methods for this approach include k Nearest Neighbors (KNN) imputation [9], Multiple Imputation (MI) techniques such as Multiple Imputation using Chained Equations (MICE) and Markov Chain Monte Carlo (MCMC) [10,11,12], and Random Forests (RFs) to replace missing data (MissForest) [13]. The last strategy combines both the spatial and temporal aspects, taking full advantage of the patterns and intricacies present within the data. For this, specific methods for WSNs have been developed, such as Data Estimation using Statistical Models (DESM) and Applying k-Nearest Neighbor Estimation (AKE). Matrix Completion (MC) methods can also be exploited here as they use correlations within one sensor and across multiple sensors but assume that the data is static, i.e., they ignore the temporal component of the data [14,15]. Other methods in this class tend to leverage deep learning to impute missing values, for example Multiple Imputation using Denoising Autoencoders (MIDA) [16] or Recurrent Neural Network (RNN)-based approaches such as Bidirectional Recurrent Imputation for Time Series (BRITS) and Multi-directional Recurrent Neural Network (M-RNN) [15,17]. For a detailed explanation of all imputation methods evaluated in this study, we refer to Section 2.3.

Previous studies have conducted various comparative analyses, assessing different datasets, classes of algorithms, setups, and types or scenarios of missingness. However, in most studies, the focus is more on multivariate time series rather than on specific WSN data. Jadhav et al. [18] compared seven imputation methods across five publicly available datasets, concluding that KNN imputation exhibited the highest performance. Similarly, Jäger et al. [19] evaluated six imputation techniques on 69 datasets, noting that random forest-based solutions generally outperformed others. Notably, their study also evaluated performance in downstream Machine Learning (ML) tasks, finding that the imputation rendered a 10–20% performance increase. Khayati et al. [20] focused on sensor time series imputation, comparing 16 recovery algorithms on six public and two synthetic datasets, including block missings, which are more reflective of WSN data characteristics. Their findings suggested that the optimal recovery method often depends on dataset-specific characteristics. Yozgatligil et al. [21] assessed six imputation techniques using Turkish State Meteorological Service data, introducing the correlation dimension technique to account for spatiotemporal dependencies in imputation evaluation. Their study indicated that the MCMC approach yielded the most favorable results.

**Table 1:** The imputation techniques that were considered in this study, together with their respective imputation strategy.

Method	Imputation Strategy
AKE [6]	WSN-specific
BRITS [17]	Deep learning
DESM [22]	WSN-specific
KNN [9]	Spatial correlations
MC [14]	Temporal and spatial correlations (static)
MCMC [12]	Spatial correlations
MICE [11]	Spatial correlations
MIDA [16]	Deep learning
MRNN [15]	Deep learning
Mean imputation	Temporal correlations
MissForest [13]	Spatial correlations
Spline [8]	Temporal correlations

In this study, we evaluated 12 imputation techniques for different artificial missing scenarios (by inducing 10%, 20%, 30%, 40%, and 50% data removal), as well as a more realistic scenario defined as “masked” missings. In this scenario, we replicated the missing patterns observed in sensors with incomplete data onto sensors with complete information, simulating a real-world missings scenario. In this way, we created a standardized scenario through which we can evaluate how effective every method is in compensating for the missing patterns. Comparisons are made based on the Root-Mean-Square Error (RMSE) and Mean Absolute Error (MAE) to assess the accuracy of the imputed values. Our study advances the existing literature by conducting a comprehensive comparison of various missing value imputation methods, employing different strategies and model types. Moreover, we analyze a genuine WSN dataset featuring a substantial sensor count (1500) and expand the assessment of these techniques from random missing values to masked missing values, offering a more realistic evaluation scenario for practical deployment.

The remainder of this paper is structured as follows: Section 2.1 introduces the CNidT project, while Section 2.2 describes the dataset collected in the project, as well as the preprocessing steps that were used. In Section 2.3, the imputation methods evaluated in this study are described, and the evaluation criteria are detailed in Section 2.4. Section 3 presents the results and discusses the implications of these results. In Section 4, we summarize our findings, list the most important insights and conclusions, and provide possible directions for future research.

## 2. Materials and Methods

### 2.1. The Curieuze-Neuzen Citizen Science Project

The dataset analyzed in this study originated from the citizen science project “Curieuze-Neuzen in de Tuin” (CNidT), which translates as “Nosy Parkers in the Garden” [23,24]. The project engaged 4400 citizen participants across the strongly urbanized and densely populated region of Flanders (Belgium, Northwestern Europe) to monitor the microclimate in their garden. The scientific objective was to quantitatively assess the impact of gardens on the local microclimate and their potential role in the mitigation of extreme weather events [25]. Initially, 50,578 citizens registered as candidates to participate in the project. From this pool of registrations, 4400 sampling locations were selected using an environmental sampling algorithm, to obtain a representative subsample that covered the range of gardens in terms of size and composition but also ensured a suitable geographical distribution across the measurement domain [26]. To this end, metadata were collected for each garden, including variables related to urbanity, garden characteristics, garden management, topography, and geography. These metadata were obtained from participants through questionnaires combined with available remote sensing data. Factor Analysis of Mixed Data (FAMD) was implemented to reduce the dimensionality of available metadata, and the selection algorithm used the first three Principal Component (PC) from FAMD coordinates to hierarchically select candidate locations, maximizing variation between gardens in the available environmental space.

The project included two six-month measurement campaigns, spanning the growing season (spring and summer) of 2021 and 2022. Citizens received a microclimate sensor device (a “lawn dagger”) that was inserted in the soil in the middle of a lawn patch in the garden. The microclimate sensor was a custom-tailored modified version of the well-established TMS logger, which provides a robust and cost-effective device to monitor temperature and soil moisture near the soil surface [27]. The TMS sensors recorded data every 15 min with three temperature sensors (DS7505 digital thermometer), one positioned at 10 cm below the soil surface, one at the soil surface, and one 12 cm above it. In addition, the TMS measures soil moisture using the time domain transmission principle in the top 15 cm of the soil [27]. The device has a large internal storage, allowing it to internally store the data collected over a period up to 10 years.

While regular TMS sensors only allow off-line data collection, a novel TMS-NB version of the instrument was specifically developed for the project, which was equipped with wireless transmission ability (collaboration between University of Antwerp, sensor development company TOMST, and telecom operator Orange Belgium). To this end, the TMS-NB was equipped with a data transfer module (BG77 Quectel with Qualcomm chipset) to send small data packages via the Narrowband Internet of Things (NB-IoT) network hosted by Orange Belgium. Measurements of temperature and soil moisture were recorded every 15 min, and the recorded data were stored in the device’s internal memory. Data collected over one day were sent as one data package via NB-IoT each day at midnight. This data package included additional metrics (e.g., battery status, signal quality) and was transmitted via the LiveObjects platform of telecom operator Orange to a relational database (MS SQL) at the University of Antwerp.

The CNidT project thus gave rise to a large WSN (>4000 nodes) that performed NB-IoT-based environmental monitoring for a period of two summers (April–September). The use of low-cost sensors and reliance on citizen input occasionally led to erroneous values or missing data points due to various factors, including random sensor malfunctioning (e.g., occasional missing data), connectivity issues (i.e., data package not sent over the NB-IoT network), as well errors and accidents by the participants (e.g., sensors damaged by kid’s play or robot lawn mowers). As sensor malfunctioning was virtually absent (<0.01% of data points) and connectivity issues could be solved by reading out the data manually after the end of the project, overall data availability ended up around 90% (see Section 2.2). Although such a percentage might be sufficient for most scientific questions, the project’s goal of reporting back to individual citizens in real time about conditions in their own garden, as well as a subsequent analysis using ML methods, makes a gap-filling exercise especially appealing.

### 2.2. Dataset and Preprocessing

**Figure 1:**
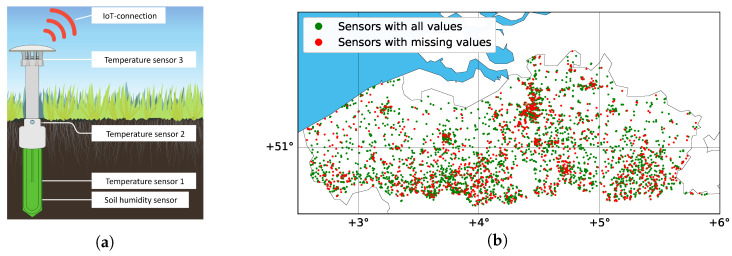
(**a**) The TMS-NB microclimate sensor was used in a large-scale citizen science project on microclimate monitoring. The sensor measures temperature at three heights, as well as soil moisture. Data transmission occurred via NB-IoT. (**b**) The WSN covered 4400 gardens across Flanders. Sensor locations are colored based on whether time series were complete (green) or had missing records (red).

A subset of the available data was used to evaluate the missing value imputation techniques. Sensor readings were retained for one six-month measurement campaign (starting on 12 April 2021, at 00:00:00 until 30 September 2021, at 23:45:00), measured every 15 min, thus providing a maximum of 16,512 records in each sensor time series (172 days of data collection times 96 readings per day). Before the final construction of the dataset, extensive manual data recovery measurements were done from the TMS-NB sensor to have as complete a dataset as possible. Furthermore, the additional metrics (e.g., battery status, signal quality) from the processed signal were also analyzed to generate as complete a sample as possible. Each sensor reading contained four data records (three temperatures at different heights and soil moisture). Data from the temperature sensor at 12 cm above the soil surface (expressed in degrees Celsius) were selected for the evaluation (and thus the remainder of our analysis), as these readings expressed the highest variability. Data series were available for 4163 sensor locations. Figure 1b illustrates the location of the sensors and whether the recorded time series for the sensor was complete or not. In total, 2978 sensors (or 71.5% of the WSN) had no missing values. Across all 4163 sensors comprising the WSN, 7.8% of records were missing. Although the missing percentage is not extremely high, about one-third of the sensors showed at least a few missing values, with some sensors missing nearly all values.

The geographical coordinates of the sensor location (uncertainty 10 m) are part of the metadata. As some methods utilize geometric distances between sensors, we calculated the haversine distance (as described by [28]) for every sensor combination as follows:

(1) $$\begin{array}{c} d_{hav}\left(x,y\right):=2r\arcsin\left(\sqrt{\sin^{2}\left(\frac{y_{lat}-x_{lat}}{2}\right)+\cos\left(x_{lat}\right)\cos\left(y_{lat}\right)\sin^{2}\left(\frac{y_{lon}-x_{lon}}{2}\right)}\right) \end{array}$$

where *x* and *y* are the coordinates of two different sensors, and *r* is the radius of the Earth (6371 km). The haversine (or great circle) distance is the angular distance between two points on the surface of a sphere. We used the haversine distance rather than the Euclidean distance to account for the Earth’s curvature given the scale of the measurement domain (Flanders region; ∼300 km).

### 2.3. Missing Value Imputation

In the literature, various methodologies for imputing missing (sensor) data exist. Below, we provide a concise overview of the methods that were considered in this study. For a more detailed explanation, we refer to their respective papers and code implementations. First, we introduce different types of missing values, after which we detail how to create suitable test datasets for imputation.

#### 2.3.1. Different Types of Missing Values

There are three types of missing data mechanisms: Missing Completely at Random (MCAR), Missing at Random (MAR), and Missing not at Random (MNAR) [29,30,31]. MCAR implies that missingness is independent of observed and unobserved data, making observed data still representative. This assumption is often strong and unrealistic. MAR means missingness is linked to observed but not unobserved data [29]. MNAR occurs when missingness is related to unobserved variables, making it the most challenging scenario to handle and non-ignorable [32].

#### 2.3.2. General Approach

To test the performance of imputation methods, we restricted ourselves to data series that had complete information, as done in other comparison studies [15,33]. From the 2978 sensors available with complete records, we selected a subset of 1500 as our basic dataset. In these complete data series, we artificially introduced data gaps that had to be resolved by imputation. Two separate approaches for missing value creation were applied.

In a first approach, we applied patterns of randomly missing values with increasing fractions of data missing (10%, 20%, 30%, 40%, and 50%) using the numpy.random.choice function [34]. This approach provides missing values that are MCAR, which is the missingness pattern that is most often used in the literature on missing value imputation [19]. Although it would also be possible to introduce MAR missing values, e.g., based on sensor location, we did not consider this option, as it is included in the masked missing scenario introduced below. Finally, MNAR missing values would manifest themselves by removing temperature values based on the actual temperature values themselves (e.g., low temperature values are removed). As our sensor operates within a range of −55 °C to 125 °C [27], but this scenario was not relevant for our dataset.

**Figure 2:**
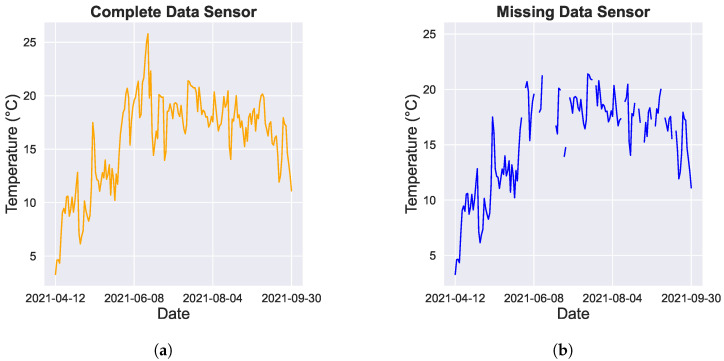
Example of missing data pattern in a representative sensor time series, which includes 15-min temperature recordings over a six-month period. (**a**) Time series for a sensor with complete data. (**b**) The same time series but with missing data artificially imposed. The missing time points are based on a mask derived from a different sensor with actual missing data.

In reality, missing data patterns are not necessarily random. In order to mimic a more realistic case, we took advantage of the missing data patterns from the sensors that actually had missing data. To this end, missing patterns were imposed from incomplete sensors onto complete sensors, thus imposing a mask with missing data. In this approach, time points for which an incomplete sensor was missing data were imposed as a mask onto a sensor with a complete data series (as illustrated in Figure 2). This approach is further referred to as the masked missing. This approach allows for a more realistic evaluation, as potential issues such as spatial or temporal block missing, for example, due to network failure or sensor failure, will be present in the data.

For all six scenarios (10%, 20%, 30%, 40%, and 50% random missing as well as the masked missing), a 2×5 nested Cross-Validation (CV) was run to obtain robust results. Nested CV involves two levels of CV loops: an outer loop and an inner loop. In the outer loop, the dataset is divided into training and testing sets using k-fold CV. Each fold of the outer loop trains the model on the training set and evaluates it on the testing set. Within each fold of the outer loop, an inner CV loop is employed where the training data are split into training and validation sets, also using k-fold CV. The inner loop is responsible for selecting the set of hyperparameters that performs best on the validation set. In our study, we used a 2×5 nested CV, i.e., we had two outer loops and five inner loops. For hyperparameter tuning, where applicable, we utilized a randomized grid search strategy. This involved exploring a predefined range of hyperparameters, as listed in Table A1, and selecting the combination that minimized the Root-Mean-Square Error (RMSE). To ensure comprehensive exploration of the hyperparameter space, we conducted tuning across 50 different hyperparameter combinations. Notably, this process was carried out each time on a randomly selected subset of 500 sensors from our dataset to ensure computational efficiency while maintaining representativeness. To facilitate transparency and reproducibility, we have included the best-performing hyperparameters used in our study, which are presented in Table A1.

**Figure 3:**
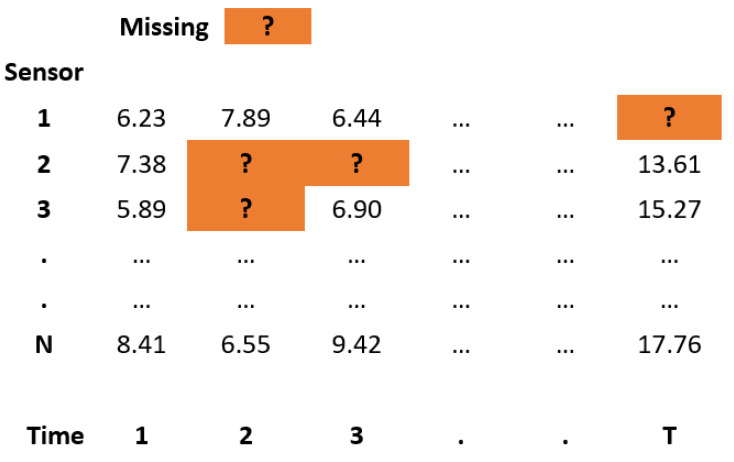
Schematic representation of a test dataset used in imputation analysis. A network of *N* sensors is providing data readings over *T* time points. Artificially induced missings are indicated by the orange fields.

In this section, we use the following notation, where a given test dataset *X* is defined as follows:

(2) $$X:=\left\{X_{nt}\right\},n\in \left\{1,\ldots ,N\right\};t\in \left\{1,\ldots ,T\right\}$$

with *N* being the number of sensors and *T* the number of time points. The imputed value for sensor *n* at time point *t* is denoted by ${\hat{X}}_{nt}$. A test dataset is schematically depicted in Figure 3. The implementation of all evaluated methods was done in Python, with the list of packages included in Table A6. We conducted our experiments on a Lenovo Thinkpad T495 2019 model with 2.3 GHz AMD Ryzen 7 PRO 3700U CPU consisting of four cores, with 4 MB of L3 cache and 16 GB of RAM.

#### 2.3.3. Mean Imputation

In this approach, missing values are filled using the arithmetic mean. Specifically, within our application, we replace missing data points from a particular sensor with the mean value of that sensor across all available time steps. Mathematically, the imputed value is expressed as shown in Equation (3), where *V* represents the set of time points with available observations for the given sensor as follows:

(3) $${\hat{X}}_{nt}=\frac{1}{\left|V\right|}\sum_{v\in V}X_{nv}.$$

Mean imputation is effective when there is limited temporal and spatial variability and when the number of missing observations for a sensor is relatively low. Because of its straightforward approach, it serves as a baseline method for comparison within our study.

#### 2.3.4. Spline Imputation

The linear spline imputation method uses temporal correlation within one sensor to impute missing values [8]. An imputed value ${\hat{X}}_{nt}$ for sensor *n* is estimated at time *t* by applying a linear interpolation based on the closest available time point in both directions, $t_{-}$ and $t_{+}$:

(4) $${\hat{X}}_{nt}=X_{nt_{-}}+\frac{X_{nt_{+}}-X_{nt_{-}}}{t_{+}-t_{-}}\left(t-t_{-}\right).$$

#### 2.3.5. K Nearest Neighbor (KNN) Imputation

The KNN method was originally developed to estimate missing values in gene expression microarray experiments, but it can be easily applied to other use cases [9]. During imputation, data points with similar features as the data point with missing values are selected. In our case, the data points are the different sensors, while the features are the values at different time points. Thus, the KNN imputation technique leverages the spatial correlation of the dataset. This method would find *k* sensors that have a value present for the missing time point, where the values of the other time points are most similar to those of the sensor with the missing value. Afterward, a weighted average of the *k* “closest” sensors is calculated to estimate the missing value. The contribution of each of the *k* sensors is weighted by its similarity to the features of the sensor with missing values, where the similarity is quantified using the Euclidean distance.

#### 2.3.6. Multivariate Imputation by Chained Equations (MICE)

The previously introduced methods all involve replacing missing values with a single estimation, disregarding the uncertainty and variability of the missingness. MI is a statistical technique used to handle missing data that generates multiple plausible imputations based on the distribution of the observed data [10,11]. Estimating multiple imputations, as opposed to just one imputation, accounts for (part of) the statistical uncertainty in the imputations [35]. MICE is an example of an MI technique and generally operates under the assumption that the missing data are MAR or MCAR [11,35,36,37]. When the data are not MAR, the application of MICE could result in biased or inaccurate estimates. The chained equations process used in MICE consists of the following steps [35,37]:

1. Make an initial guess about the missing values using a simple imputation method, such as mean imputation.
2. Set the missing values for one feature *f* back to missing. The observed values for *f* are then regressed using (all) other features in the dataset.
3. Make a prediction for the missing values of *f* using the regression model from the previous step.
4. Repeat steps 2 and 3 for all features that contain missing values. At the end of this step, all features with missing values have been imputed.
5. Repeat steps 2, 3, and 4 for a number of cycles and update the imputations in each cycle.

The number of cycles can be chosen by the user and is task-dependent. The final imputation is the imputation found in the final cycle [35].

#### 2.3.7. Markov Chain Monte Carlo (MCMC) Imputation

Another MI technique is MCMC [12], based on the Bayesian framework. In essence, MCMC leverages the principles of a Markov Chain and Monte Carlo simulation to approximate missing values by iteratively sampling from a probability distribution. The main focus is finding the desired posterior distribution defined by a set of parameters θ, from which the unobserved values $X_{u}$ can be predicted using the conditional density of the observed observations $X_{o}$ [12,38]. The method starts from an initial, plausible approximation of the missing readings $X_{u}$. In the next step, the MI technique starts. Given certain parametric assumptions, the $\theta^{i}$ can be estimated from the posterior distribution $f(\theta^{i}|X_{o},X_{u})$, with $\theta^{i}$ being the estimated parameter values in step *i*. In a second step, the predictive distribution can be used to obtain the improved predicted values $X_{u}^{i}$ at iteration *i*.

(5) $$X_{u}^{i+1}∼f\left(X_{u}|\theta^{i},X_{o}\right)$$

In the next step, the θ parameter values can again be estimated from the complete data posterior distribution using the newly acquired values.

(6) $$\theta^{i+1}∼f\left(\theta |X_{o},X_{u}^{i+1}\right)$$

These last two steps are iteratively executed until gradually converging to the true distribution. Due to the sequential sampling from two distributions, a Markov Chain is made, and the use of simulations renders the MCMC name [12,38,39]. MCMC imputation offers several advantages, including the ability to handle complex data structures and missingness patterns, as well as the flexibility of incorporating prior knowledge or constraints. The technique tends to be computationally intensive for large datasets, and it often requires careful tuning.

#### 2.3.8. Matrix Completion (MC) Imputation

Another imputation technique based on iterative MI is matrix completion, based on [14]. The method uses the spatial and temporal correlations of the data to impute missing values. The main idea of MC is to handle missing values in a data matrix by imputing them with estimates based on the observed values and the low-rank structure of the data matrix. More precisely, missing readings get replaced iteratively with those obtained from a soft-thresholded singular value decomposition [14]. First, a singular value decomposition is applied to the incomplete matrix with soft-thresholding, where the nuclear norm of the matrix is used as a regularizer. In the next step, the modified singular value matrices are used to reconstruct the data matrix. Then, these two steps are iterated until convergence of the imputed values is reached. The matrix completion technique as discussed by [14] is well-suited for situations where the data matrix can easily be approximated by a lower-rank matrix, rendering an effective solution to the missing value problem for large and sparse matrices.

#### 2.3.9. Data Estimation Using Statistical Model (DESM) Imputation

Similar to previously discussed methods, DESM uses temporal and spatial correlations between sensors to impute missing values [22]. The method is specifically developed for WSNs, with the sensor data specific characteristics in mind. More specifically, DESM relies on historical values of the sensor for which a value is missing (sensor *n*), as well as the values of the sensor spatially located the closest (sensor *m*), with the requirement that the latter sensor does not have missing values around the time point that needs to be imputed. Missing values are then estimated according to

(7) $${\hat{X}}_{nt}=\left(1-\alpha \right)\hat{Y}+\left(\alpha \right)\hat{Z}.$$

In Equation (7), $\hat{Y}$ is the imputed value at the previous time point ${\hat{X}}_{n(t-1)}$, and $\hat{Z}$ is defined as follows:

(8) $$\hat{Z}=X_{n(t-1)}\left(1+\frac{X_{mt}-X_{m(t-1)}}{X_{m(t-1)}}\right).$$

DESM leverages both the temporal influence of one sensor on itself, included in $\hat{Y}$, as well as the spatial attributes of the other sensors (in this case *m*), represented by $\hat{Z}$, to impute the missing readings. The α in Equation (7) is the Pearson correlation coefficient between two sensors $X_{n}$ and $X_{m}$, which serves as a weight parameter that evaluates the effects of $\hat{Y}$ and $\hat{Z}$ on the estimated value. Equation (8) is based on the assumption that the data collected by sensors $X_{n}$ and $X_{m}$ are approximately similar, as they are spatially close to each other.

#### 2.3.10. Applying k Nearest Neighbor Estimation (AKE) Imputation

Sensors that are located in close spatial proximity to other sensors will yield very similar measurements, which means that it is possible to impute missing values based on the neighboring sensor values for the same time point. As the exact functional relationship between two nearby sensors is unknown, the AKE method assumes that this relationship can be approximated linearly in a short time period [6]. Under this assumption, we can estimate missing values $X_{nt}^{m}$ from a neighbor sensor $X_{mt}$ for any time *t* using linear regression

(9) $${\hat{X}}_{nt}^{m}=\alpha +\beta X_{mt},$$

where α and β are estimated using all non-missing $\left(X_{nt},X_{mt}\right)$ pairs. In total, *k* linear regression models will be fitted for every sensor, where *k* is a tunable hyperparameter. To obtain an imputed value, we have to combine the estimations from all *k* neighboring sensors. While using the arithmetic mean of all imputations is a valid option, this would disregard the strength of the linear correlation between two sensors. For this reason, AKE uses a weighted average of all *k* estimated values

(10) $${\hat{X}}_{nt}=\sum_{m=1}^{k}w_{nm}\;\cdot \;{\hat{X}}_{nt}^{m},$$

where $w_{nm}$ is the weight, for which $0\leq w_{nm}\leq 1$ and $\sum_{m=1}^{k}w_{nm}=1$. As we can assess the performance of a linear regression by using the determination coefficient $r^{2}$, we define the weight $w_{nm}$ as the normalized determination coefficient as follows:

(11) $$w_{nm}=\frac{r_{nm}^{2}}{\sum_{j=1}^{k}r_{nj}^{2}}.$$

#### 2.3.11. MissForest Imputation

The MissForest method is a non-parametric imputation method that uses an RF to impute missing values [13]. To start, an initial guess is made for the missing values, using mean imputation or another imputation method. Afterward, the RF is trained on all data, including the initial guess for the missing values. The trained RF is then used to impute the missing values again. This procedure is repeated for a fixed number of iterations or until a stopping criterion is reached, whichever comes first. The stopping criterion is met as soon as the difference between the previously imputed values (${\hat{X}}^{old}$) and the newly imputed values (${\hat{X}}^{new}$) increases for the first time, i.e., the imputation has converged. The difference Δ is defined as follows, with *p* being the total number of missing values:

(12) $$\Delta =\frac{\sum_{i=1}^{p}{\left({\hat{X}}_{i}^{new}-{\hat{X}}_{i}^{old}\right)}^{2}}{\sum_{i=1}^{p}{\left({\hat{X}}_{i}^{new}\right)}^{2}}.$$

Due to the use of random forests, the method is relatively robust against outliers; however, it can become computationally expensive on large datasets.

#### 2.3.12. Multiple Imputation Using Denoising Autoencoders (MIDA)

MIDA is another MI technique that uses overcomplete Denoising Autoencoders (DAEs) to impute missing values [16]. An overcomplete DAE is a DAE where the input data are projected to a higher-dimensional subspace, from which the missing values are recovered. The input layer has *T* nodes, assuming the data have *T* features (time points in our use case). Then, each successive hidden layer adds Θ nodes, where Θ is a tunable hyperparameter. This is done for *j* encoding layers, after which j−1 decoding layers are added, which decrease the number of nodes from T+jΘ to *T* for the output layer. Empirically, Θ=7 and j=3 have been found to be a good choice, but both of these parameters can be seen as tunable hyperparameters [16]. The MI part of MIDA is established by initializing the model with a different set of random weights in multiple runs, thereby providing multiple predictions. By leveraging the representational learning capabilities of denoising autoencoders, MIDA can capture the underlying patterns in the data, thus potentially generating more realistic results. However, these results heavily depend on the quality and similarity between the unknown and known observations in the training data. Furthermore, the use of autoencoders and MI also make this method computationally expensive.

#### 2.3.13. Bidirectional Recurrent Imputation for Time Series (BRITS)

There are several methods based on an RNN for missing value imputation, such as BRITS [17]. The imputation method tries to learn the missing values in a bidirectional recurrent dynamical system, without any specific assumptions [17]. The method was originally developed for missing value imputation in multiple correlated time series, which we extend to the WSN framework. In BRITS, an RNN is used directly for predicting missing values, meaning that missing values are regarded as variables of the bidirectional RNN graph, leveraged in the back-propagation of the neural network. This approach ensures that missing values receive delayed gradient updates in both the forward and backward directions, with consistency constraints. The model architecture can also be leveraged for simultaneous regression or classification jointly in one graph, rather than pure missing imputation. This can mitigate a part of the error propagation in subsequent modeling tasks. The main advantages of the BRITS imputation method is the application to general missing settings, as well as the ability to handle correlated time series and nonlinear dynamics within the data.

#### 2.3.14. Multi-Directional Recurrent Neural Network (M-RNN) Imputation

Closely related to BRITS is M-RNN, as introduced by [15]. M-RNN imputes values both within and across data-streams, thus both in a temporal and spatial fashion. The original method was developed for clinical applications, yet it can be easily applied to other scenarios. The imputation technique contains both an interpolation block (temporal) and an imputation block (spatial), which are trained simultaneously. The interpolation block uses an adjusted bi-directional RNN with a lagged timing for the inputs into the hidden layers in the forward direction and advanced in the backward direction [15]. The imputation block is then a fully connected neural network with dropout. Similarly to BRITS, the method can also be used for a subsequent modeling task directly. Notably, the M-RNN tends to be less affected by both the quantity and specific nature of missing data.

### 2.4. Empirical Evaluation

To compare the performance of the different methods, it is important to have a predetermined set of performance metrics. In this study, we use the RMSE and MAE to assess the accuracy of the imputed values. Based on previous studies, we also evaluate the percentage of cases in which a missing value can be estimated, i.e., the Prediction Coverage Error (PCE), as defined in Equation (13) [40,41].

(13) $$\mathrm{PCE}=\frac{\mathrm{number}\;\mathrm{of}\;\mathrm{successful}\;\mathrm{imputations}}{\mathrm{number}\;\mathrm{of}\;\mathrm{missings}}\times 100\%$$

The PCE is necessary to be able to interpret the RMSE and MAE fairly; indeed, the RMSE and MAE ignore missing data points that the model was unable to impute. As a result, the RMSE and MAE might be underestimated for some methods that are not able to impute all samples, resulting in an overstatement of the model performance. For these cases, the PCE provides additional context.

## 3. Results and Discussion

### 3.1. Random Missings

**Figure 4:**
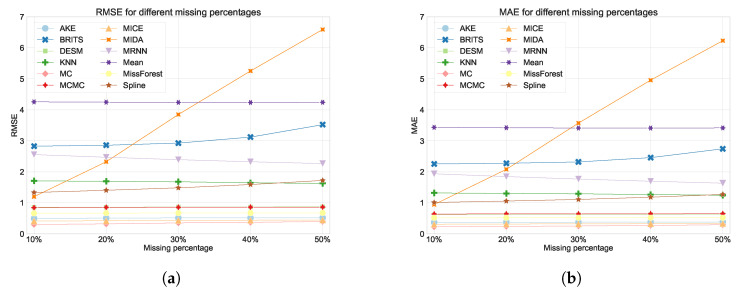
(**a**) The RMSE for all models for different degrees of missingness. (**b**) The MAE for all models for different degrees of missingness.

**Figure 5:**
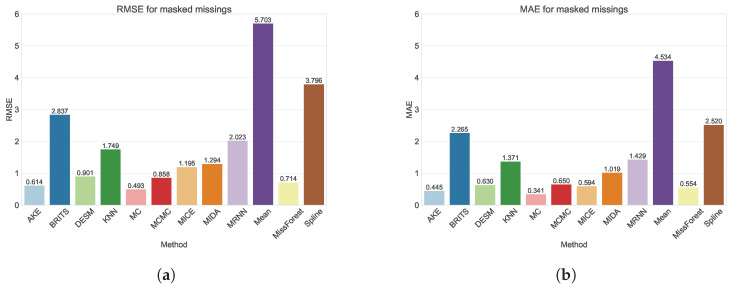
(**a**) The RMSE for all models for the masked data. (**b**) The MAE for all models for the masked data.

The performance of the various imputation methods, evaluated using the metrics defined in Section 2.4, is depicted in Figure 4 and Figure 5. Exact values for the RMSE, MAE, PCE, and execution times are detailed in Table A2, Table A3, Table A4 and Table A5. As can be seen in Figure 4a,b and Table A2 and Table A3 the MC imputation method achieves the best performance (smallest RMSE and MAE) for all degrees of missingness. Generally, all methods consistently outperform the baseline mean imputation, except for the MIDA method, where performance diverges for higher degrees of missingness (see Section 3.5). In the results, a noticeable trend is observed where methods considering the spatial features of the data generally outperform others. Methods such as MissForest and MCMC obtain a good performance and even outperform DESM, which is specifically tailored for WSNs problems. AKE, another WSN-specific method, has a very good performance and is only outperformed by MC and MICE. For nearly all methods, the performance gets worse with increasing degrees of missingness, which is expected and is also commonly observed in the literature [15,41]. A notable exception to this rule is M-RNN, as its performance remains stable with increasing degrees of missingness. Although unexpected, we consider this result to be less significant, as the difference is relatively small, and the absolute performance of M-RNN is among the worst for our specific use case. In addition, M-RNN can efficiently handle higher degrees of missingness, explaining the result.

### 3.2. Masked Missings

As random missings do not accurately represent real-life missing scenarios, we also evaluated all methods on a realistic missings dataset obtained by creating masks from real, observed missing patterns. From Figure 5a,b and Table A2 and Table A3, we can conclude that the MC method performs best, as was also the case in the random missing scenario. Similarly, AKE, DESM, MCMC, and MissForest are again among the top performing methods, indicating the better performance of spatial methods on our dataset. As expected, the performance in the masked missings scenario is generally worse than for random missings, as so-called “block” missings frequently cause a lack of “nearby” data points, which are often used to impute missing values. In particular, the performance of spline imputation and MICE is significantly worse in the masked scenario. This finding was expected for spline imputation, as it applies a linear interpolation based on the closest time points surrounding the missing value, which will often be missing itself in the case of block missings. For MICE, this can be attributed to a worse convergence of the model due to a poor initial guess. More specifically, the initial guess for the MICE algorithm was made using mean imputation. From Table A2 and Table A3, we can clearly see that mean imputation performs significantly worse for masked missings when compared to its performance for random missings and thus probably did not provide an accurate initial guess.

### 3.3. Prediction Coverage Error

**Figure 6:**
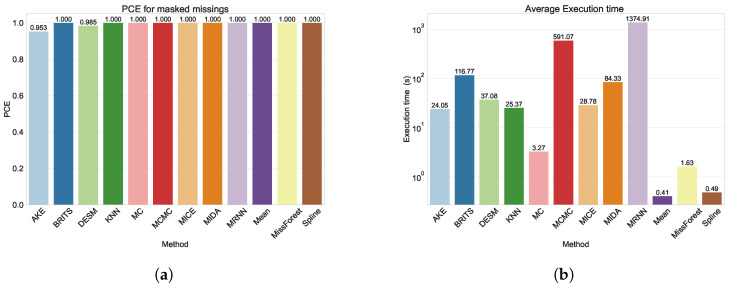
(**a**) The PCE for all models for the masked data. (**b**) The execution time (on a logarithmic scale) for all models, averaged over all degrees of missingness, including masked missings.

We should, however, interpret the results for AKE and DESM in the masked missings scenario with caution. In fact, Figure 6a and Table A4 show that these methods do not achieve a PCE of 1, meaning that they were unable to impute all missing values. Indeed, AKE and DESM were only able to impute 95.3% and 98.5% of the missing values, respectively. As AKE requires at least one sensor (in a group of *k* nearby sensors) to have an observed value in the given time step and DESM requires one sensor to have no missing values around the considered time step, this is likely attributable to large-scale network outages. Indeed, these outages result in spatially nearby points failing simultaneously. Although the number of unfilled missings is quite small, an end user might opt to address the remaining gaps by employing a straightforward imputation method such as mean imputation.

### 3.4. Execution Time

A final criterion to consider when evaluating the imputation methods is the time needed to train the method (if necessary) and make the imputations, which is combined in the execution time. In Figure 6b and Table A5, the execution time averaged over all degrees of missingness and masked missings is shown. As expected, the deep learning-based methods (M-RNN, MIDA, and BRITS) were very computationally intensive to train. The MCMC method was also very computationally expensive, which was expected, as the authors state that this is the case for large datasets [12]. Finally, even though the MC method is specifically designed for large matrices, it is interesting to note that it achieved a very small execution time while also providing the best imputed values in both missing scenarios.

### 3.5. Discussion

Within our empirical evaluation, spatial methods tend to outperform others across all imputation scenarios. This can be attributed to the large number of sensors as well as the spatial proximity of the sensors. Furthermore, the evaluated time steps were limited, thus impacting the performance. In general, these results are in line with the literature. However, the deep learning-based methods exhibited poor performance on our dataset, regardless of the missing pattern. We attribute this to several factors. First, the relatively short time series considered in these data may limit the ability of certain deep learning-based methods, such as BRITS, to accurately capture temporal dependencies and patterns. Additionally, most deep learning applications tend to evaluate datasets with either a higher frequency of measurements or a higher number of measured objects or sensors. Furthermore, our dataset considers the behavior of small microclimates, in which local effects may cause temperature peaks that do not appear for other sensors, which complicates generalization to other sensors. Nevertheless, deep learning-based methods do exhibit good results for other datasets and should therefore not be ignored as a possible imputation technique [15,16,17]. They also offer the added advantage of making supplementary predictions using the acquired structure [15,17] or by training new models based on the already learned weights. For datasets where deep learning-based methods perform very well or at least comparably to other methods, this added benefit could be the deciding factor.

## 4. Conclusions

During the last decade, sensors have become increasingly important across scientific fields and industries. Unfortunately, sensor data often contain missing values, which can significantly hamper the interpretation and possible analysis of the collected data. Consequently, the importance of methods capable of imputing these missing values with accurate estimates has grown considerably. In this study, we conducted a comparison of twelve imputation methods on a unique environmental microclimate monitoring dataset collected by the CNidT citizen science project. We extend the current literature by providing an extensive comparison of different missing value imputation methods originating from different backgrounds and imputation strategies. In addition, our work considers a real WSN dataset with a large number of sensors (1500), which is uncommon in the literature. Furthermore, we extend the evaluation of the evaluated techniques from random missings to masked missings, which provides a highly realistic evaluation scenario for practical implementations.

We evaluated the imputation methods for two different missing patterns: random missings, with the degree of missingness ranging from 10% to 50%, and masked missings, which were obtained using realistic missing value patterns. For all missing patterns, the MC method outperformed all other methods. MissForest and MCMC also performed relatively well in both scenarios, while MICE only achieved good results for random missings. The methods that are designed for WSNs specifically also performed well in both scenarios; however, they were not able to provide imputations for all missing values in the masked missings scenario. Finally, the deep learning-based methods, M-RNN, MIDA, and BRITS, performed poorly for both missing patterns, which can be attributed to the characteristics of our dataset. We can conclude from the results obtained that the methods that exploit spatial correlations within the dataset tend to perform better than the other methods. This can be explained by the relatively small distance between sensors, as well as the granularity of the temporal component. Moreover, since the data encompassed the period from April to September, temperatures predominantly experienced an upward trajectory, making it challenging to discern a clear trend in the temporal aspects of the data. These results can be extrapolated to similar scenarios where the number of sensors is high and densely distributed with a comparable length of time. The success of methods such as MC, MissForest, and MCMC, particularly in capturing spatial correlations within the dataset, suggests that they would generalize well to such environments. Despite challenges posed by masked missing values, these methods still demonstrated robust performance, implying their potential applicability in scenarios with complex missing data patterns.

Future research can expand upon our study with a more detailed assessment of (other) methods on different datasets. More specifically, different numbers of sensors and temporal granularity can be evaluated to more clearly identify the impact of these dataset specific features on the evaluated models. This can aid in the identification of a general best imputation technique across different WSNs. Furthermore, in future studies concerning missing data imputation for WSNs, additional features of the sensors or locations can be used to address missing values, such as the type of microclimate location, or other measured variables, such as the humidity in our specific use case. Also, the development of novel WSN-specific methods that efficiently exploit all structures (spatial and temporal) that are available in the data, carry significant potential. For example, a method could use an MI approach by first imputing all missing values using temporal correlations and subsequently using these imputations to obtain a more accurate spatial imputation, or vice versa. Additionally, cost-sensitive methods for missing value imputation can be evaluated, where over- or underestimations of the actual value can be penalized more heavily. Moreover, the evaluation of the temporal and spatial granularity and its impact on the imputation performance for various methods could be a valuable addition. Finally, our comparative study focuses on daily temperature values, whereas it may be interesting to evaluate it per 15-min interval or hourly and assess the imputation performance.

In conclusion, we were able to successfully impute missing values in our unique environmental monitoring dataset and provided guidelines for researchers who want to impute missing values in a similar dataset. Ultimately, we found that the best method to impute missing values is often dataset-specific and should be identified using a set of artificially induced missings, preferably both randomly generated and based on a realistic missing pattern.

## Data Availability

Publicly available datasets were analyzed in this study. The data can be found at: https://www.vlaanderen.be/datavindplaats/catalogus/curieuzeneuzen-in-de-tuin (accessed on 27 February 2024). Data can also be found in the SoilTemp database available at: https://www.soiltempproject.com/ [7]. Code for the replication of our study is available on GitHub: https://github.com/ThomasDecorteUA/Missing_Imputation_Sensors (accessed on 5 April 2024).

## Abbreviations

The following abbreviations are used in this manuscript:

AKE	Applying k-Nearest Neighbor Estimation
BRITS	Bidirectional Recurrent Imputation for Time Series
CNidT	Curieuze-Neuzen in de Tuin
CV	Cross-Validation
DAE	Denoising Autoencoder
DESM	Data Estimation using Statistical Models
FAMD	Factor Analysis of Mixed Data
IoT	Internet of Things
KNN	k Nearest Neighbors
M-RNN	Multi-directional Recurrent Neural Network
MAE	Mean Absolute Error
MCMC	Markov Chain Monte Carlo
MC	Matrix Completion
MCAR	Missing Completely at Random
MAR	Missing at Random
MICE	Multiple Imputation using Chained Equations
MIDA	Multiple Imputation using Denoising Autoencoders
MI	Multiple Imputation
MNAR	Missing not at Random
ML	Machine Learning
NB-IoT	Narrowband Internet of Things
PC	Principal Components
PCE	Prediction Coverage Error
RNN	Recurrent Neural Network
RF	Random Forest
RMSE	Root-mean-square Error
WSN	Wireless Sensor Network

## Appendix A. Hyperparameter Optimization and Results

**Table A1:** The hyperparameter grid search space for each evaluated model.

Method	Hyperparameter Search Space	Optimal Hyperparameter Value
AKE [6]	*k*: [2–25]	15
	timesteps: [5–30],	18
BRITS [17]	hidden dimensions: [5–15],	8
	learning rate: [0.0001,0.001,0.01,0.1]	0.001
KNN [9]	N neighbors: [2–25]	5
MICE [11]	N nearest features: [2–25]	24
MIDA [16]	Θ: [5,6,7,8,9]	7
	sequence length: [5–30],	5
MRNN [15]	hidden dimensions: [5–25],	7
	learning rate: [0.0001,0.001,0.01,0.1]	0.01

**Table A2:** The RMSE scores for all imputation models, for different degrees of missingness and masked missings. The best performances are indicated in bold.

Method	10%	20%	30%	40%	50%	Masked
AKE [6]	0.499	0.508	0.514	0.516	0.520	0.614
BRITS [17]	2.829	2.859	2.928	3.122	3.523	2.837
DESM [22]	0.857	0.862	0.866	0.870	0.875	0.901
KNN [9]	1.711	1.695	1.680	1.648	1.628	1.748
MC [14]	**0.304**	**0.322**	**0.354**	**0.372**	**0.398**	**0.493**
MCMC [12]	0.847	0.856	0.858	0.861	0.864	0.858
MICE [11]	0.414	0.422	0.437	0.440	0.448	1.195
MIDA [16]	1.200	2.326	3.850	5.256	6.593	1.294
MRNN [15]	2.558	2.472	2.394	2.326	2.268	2.023
Mean Imputation	4.259	4.250	4.241	4.239	4.245	5.703
MissForest [13]	0.664	0.669	0.673	0.674	0.675	0.714
Spline [8]	1.335	1.406	1.486	1.592	1.724	3.796

**Table A3:** The MAE scores for all imputation models, for different degrees of missingness and masked missings. The best performances are indicated in bold.

Method	10%	20%	30%	40%	50%	Masked
AKE [6]	0.370	0.375	0.376	0.379	0.384	0.444
BRITS [17]	2.255	2.277	2.321	2.460	2.744	2.265
DESM [22]	0.617	0.621	0.622	0.627	0.631	0.630
KNN [9]	1.320	1.304	1.293	1.267	1.254	1.371
MC [14]	**0.226**	**0.240**	**0.255**	**0.272**	**0.294**	**0.341**
MCMC [12]	0.640	0.645	0.644	0.648	0.650	0.650
MICE [11]	0.311	0.318	0.322	0.328	0.335	0.594
MIDA [16]	0.946	2.082	3.574	4.959	6.233	1.019
MRNN [15]	1.938	1.848	1.768	1.698	1.635	1.429
Mean Imputation	3.436	3.424	3.414	3.413	3.418	4.534
MissForest [13]	0.519	0.520	0.520	0.522	0.523	0.554
Spline [8]	1.013	1.059	1.110	1.183	1.272	2.520

**Table A4:** The PCE scores for all imputation models, for different degrees of missingness and masked missings. The best performances are indicated in bold.

Method	10%	20%	30%	40%	50%	Masked
AKE [6]	**1.000**	**1.000**	**1.000**	**1.000**	**1.000**	0.953
BRITS [17]	**1.000**	**1.000**	**1.000**	**1.000**	**1.000**	**1.000**
DESM [22]	**1.000**	**1.000**	**1.000**	**1.000**	0.999	0.985
KNN [9]	**1.000**	**1.000**	**1.000**	**1.000**	**1.000**	**1.000**
MC [14]	**1.000**	**1.000**	**1.000**	**1.000**	**1.000**	**1.000**
MCMC [12]	**1.000**	**1.000**	**1.000**	**1.000**	**1.000**	**1.000**
MICE [11]	**1.000**	**1.000**	**1.000**	**1.000**	**1.000**	**1.000**
MIDA [16]	**1.000**	**1.000**	**1.000**	**1.000**	**1.000**	**1.000**
MRNN [15]	**1.000**	**1.000**	**1.000**	**1.000**	**1.000**	**1.000**
Mean Imputation	**1.000**	**1.000**	**1.000**	**1.000**	**1.000**	**1.000**
MissForest [13]	**1.000**	**1.000**	**1.000**	**1.000**	**1.000**	**1.000**
Spline [8]	**1.000**	**1.000**	**1.000**	**1.000**	**1.000**	**1.000**

**Table A5:** The execution times for all imputation models, for different degrees of missingness and masked missings. The lowest execution times are indicated in bold.

Method	10%	20%	30%	40%	50%	Masked
AKE [6]	13.1	18.0	28.1	26.5	46.2	12.4
BRITS [17]	52.4	81.7	103.0	123.6	303.8	36.1
DESM [22]	10.2	19.8	44.2	47.0	92.4	8.8
KNN [9]	15.3	21.7	27.6	34.2	41.6	11.8
MC [14]	1.5	2.0	3.9	3.7	6.2	2.2
MCMC [12]	537.0	552.1	572.3	567.6	881.5	435.9
MICE [11]	23.5	25.7	32.6	25.8	41.6	23.4
MIDA [16]	70.1	71.2	106.0	81.5	108.6	68.6
MRNN [15]	1180.5	1193.6	1640.9	1278.7	1811.7	1144.0
Mean Imputation	**0.3**	**0.3**	**0.5**	**0.4**	**0.6**	**0.3**
MissForest [13]	1.3	1.4	1.8	1.6	2.3	1.3
Spline [8]	0.4	0.4	0.7	0.4	0.7	0.4

## Appendix B. Package Versions

**Table A6:** The versions of the Python packages used in the project.

Package	Version	Reference
python	3.11.5	[42]
scikit-learn	1.3.2	[43]
fancyimpute	0.7.0	[44]
geopy	2.4.1	[45]
keras	2.12.0	[46]
missforest	2.3.1	[13]
numpy	1.23.5	[34]
pandas	2.1.4	[47]
scipy	1.11.4	[48]
seaborn	0.13.1	[49]
tensorflow	2.12.0	[50]
tensorflow-probability	0.20.0	[50]
torch	2.1.2	[51]
